# Endoscopic partial closure followed by adequate drainage for treating delayed perforation caused by duodenal endoscopic submucosal dissection: A case report: Erratum

**DOI:** 10.1097/MD.0000000000017593

**Published:** 2019-10-04

In the article, “Endoscopic partial closure followed by adequate drainage for treating delayed perforation caused by duodenal endoscopic submucosal dissection: A case report”,^[1]^ which appears in Volume 98, Issue 22 of *Medicine*, Drs. Liansong Ye and Yiping Wang are cofirst authors.
